# First Impressions of a Medical Examiner

**Published:** 1895-04

**Authors:** Edward Cranch

**Affiliations:** Erie, Pa.; Member of the State Board of Medical Examiners, representing the Homœopathic Medical Society of Pennsylvania


### FIRST IMPRESSIONS OF A MEDICAL EXAMINER.

EDWARD CRANCH, M. D., ERIE, PA.

Member of the State Board of Medical Examiners, representing the Homœopathic Medical Society of Pennsylvania.

The new medical license law of Pennsylvania has been in operation just one year. The New York law was taken as a guide, with three separate boards, representing the three regular schools of medicine—that is, those having regularly chartered colleges and regular courses of study. Instead of the Regent's

Board, which Pennsylvania has not, a Medical Council was created, composed of the Lieutenant Governor, Attorney General, Secretary of Internal Affairs, Superintendent of Public Instruction, President of the State Board of Health, and the Presidents of the three Examining Boards. Each board submits a list of questions, from which the Council selects a certain number, uniform for all the boards in the departments of Anatomy, Physiology, Pathology, Diagnosis, Hygiene, Surgery, Obstetrics, and Chemistry, differing only in Materia Medica, Therapeutics, and Practice.

All physicians intending to practice in this State are required to make application to the Council, which will inquire fully into character and education, receive fees, and issue permits to appear for examination before whichever board is desired.

After July 1st, 1895, evidence of four years' study in medicine will be required.

The first examinations under the new law were held in June, and succeeding ones in October and February, each one lasting three days and a half, being divided into seven papers, allowing three hours for each set of ten questions. The question papers from the Council were given out only at the hour allotted for each subject, and the examinations were conducted with every available precaution.

No one was examined who was not already a graduate, so there should have been no failures, especially as a general average of seventy-five per cent. was all that was needed to pass. In spite of all conditions, over five per cent. of all schools failed utterly, and many papers, even among those finally passed, showed a deplorable lack of general and special culture. Every allowance was made for uncertainty of expression, where the writer apparently knew what the question really meant; yet, in such practical subjects as the sounds of the heart, the anatomy of the thigh, the bones of the forearm, the functions of the facial nerve, the processes of respiration, the pathology of Bright's disease, the phenomena of dialyis, the nature of hydrogen peroxide, the action of Nux-vomica, etc., the answers too often indicated a total lack of information, or such want of comprehension as made them valueless. And all this was the work of those who were regularly graduated by learned faculties, from regularly chartered colleges, and were therefore all regular physicians! In the opinion of the examiners, the new law has been fully justified, and the warning to all colleges and future students is unmistakable. In time, as the representatives of former carelessness die or resign, the benefits of the new law will be tangibly apparent, and the standard of medical education will be effectually raised; so that even the rural districts will be sure of competent physicians.

Pennsylvania needs such a law to protect it from being the dumping-ground of the rejected of other States. All physicians need the law to protect their own interests by excluding ignorance and incapacity from the ranks, making the profession again what it once was, learned and liberal.

What shall be said of "medical sectarianism," which was to be crushed out by these Examining Boards, which the American Medical Association resolved to urge upon all States for that purpose?

Homœopaths and Eclectics, debarred from presenting their researches and results of practice in the journals of the majority, and refused any hearing in their societies, were forced, if they would avoid extinction by those who had prejudged them without scientific investigation, to assert before the legislatures their claim for equal rights, on the ground of equal education and culture, and generally with success, for legislators are disposed to see fair play and freedom of speech ensured to all.

If, however, the present law is left undisturbed, it will eventually see the cessation of medical sectarianism by demonstrating the real unity of all schools on most subjects, and by stimulating mutual investigation of the subjects on which they differ. At present, mainly through fear of ridicule, the bulk of the profession remains ignorant of the real meaning and application of the greatest truth of medical science—a statement of natural law, and not an "exclusive dogma"—"Similia similibus curantur," and its inevitable corollaries, the minimized dose and the single remedy. When this law is studied and taught in all colleges, and the fact recognized that it takes at least two years more study to be a good homœopath than to rest in the shadow of old physic, then the mission of the homœopathic school will have been accomplished, and it will cease to have to hold a separate existence and course, for all will know it and use its teachings. True eclecticism will then flourish, for all will strive to get the best of everything. All must realize that it is never too late to learn, and that ridicule and ostracism are poor arguments.

